# Exploring the causal effect between lipid-modifying drugs and idiopathic pulmonary fibrosis: a drug-target Mendelian randomization study

**DOI:** 10.1186/s12944-024-02218-6

**Published:** 2024-08-01

**Authors:** Gexiang Cai, Jingjing Liu, Mengsi Cai, Lianyou Shao

**Affiliations:** Department of Respiratory and Critical Care Medicine, The First Affiliated Hospital of Wenzhou Medical University, Wenzhou, Zhejiang China

**Keywords:** Idiopathic pulmonary fibrosis, Lipids, Drug-target Mendelian randomization, PCSK9, Summary-data-based Mendelian randomization

## Abstract

**Background:**

Idiopathic pulmonary fibrosis (IPF) is a respiratory disorder of obscure etiology and limited treatment options, possibly linked to dysregulation in lipid metabolism. While several observational studies suggest that lipid-lowering agents may decrease the risk of IPF, the evidence is inconsistent. The present Mendelian randomization (MR) study aims to determine the association between circulating lipid traits and IPF and to assess the potential influence of lipid-modifying medications for IPF.

**Methods:**

Summary statistics of 5 lipid traits (high-density lipoprotein cholesterol, low-density lipoprotein cholesterol, triglyceride, apolipoprotein A, and apolipoprotein B) and IPF were sourced from the UK Biobank and FinnGen Project Round 10. The study’s focus on lipid-regulatory genes encompassed PCSK9, NPC1L1, ABCG5, ABCG8, HMGCR, APOB, LDLR, CETP, ANGPTL3, APOC3, LPL, and PPARA. The primary effect estimates were determined using the inverse-variance-weighted method, with additional analyses employing the contamination mixture method, robust adjusted profile score, the weighted median, weighted mode methods, and MR-Egger. Summary-data-based Mendelian randomization (SMR) was used to confirm significant lipid-modifying drug targets, leveraging data on expressed quantitative trait loci in relevant tissues. Sensitivity analyses included assessments of heterogeneity, horizontal pleiotropy, and leave-one-out methods.

**Results:**

There was no significant effect of blood lipid traits on IPF risk (all *P*＞0.05). Drug-target MR analysis indicated that genetic mimicry for inhibitor of NPC1L1, PCSK9, ABCG5, ABCG8, and APOC3 were associated with increased IPF risks, with odds ratios (ORs) and 95% confidence intervals (CIs) as follows: 2.74 (1.05–7.12, *P *= 0.039), 1.36 (1.02–1.82, *P *= 0.037), 1.66 (1.12–2.45, *P *= 0.011), 1.68 (1.14–2.48, *P *= 0.009), and 1.42 (1.20–1.67, *P *= 3.17×10^-5^), respectively. The SMR method identified a significant association between PCSK9 gene expression in whole blood and reduced IPF risk (OR = 0.71, 95% CI: 0.50–0.99, *P* = 0.043). Sensitivity analyses showed no evidence of bias.

**Conclusions:**

Serum lipid traits did not significantly affect the risk of idiopathic pulmonary fibrosis. Drug targets MR studies examining 12 lipid-modifying drugs indicated that PCSK9 inhibitors could dramatically increase IPF risk, a mechanism that may differ from their lipid-lowering actions and thus warrants further investigation.

**Supplementary Information:**

The online version contains supplementary material available at 10.1186/s12944-024-02218-6.

## Introduction

Idiopathic pulmonary fibrosis (IPF) is a progressive and chronic disorder of unknown origin, affecting an estimated 3 million individuals globally [1]. The condition is characterized by a high mortality rate, with a median survival time of approximately 3.8 years following diagnosis [2, 3]. The pathophysiology of IPF is highly intricate, encompassing alterations in genetic factors, cellular signaling, apoptosis, autophagy, and additional processes [1]. This multifaceted nature significantly complicates the development of effective therapeutic strategies for IPF. Currently, nintedanib and pirfenidone are the only medications approved by the Food and Drug Administration for IPF treatment [4, 5]. Although these treatments can mitigate symptoms, they do not address the underlying disease, and lung transplantation may become necessary for patients to extend their lifespan [6, 7].

Current research indicates that metabolic changes play a pivotal role in the fibrosis process [8]. As a lipid-rich organ, the lung's lipid metabolism and its regulation are essential for normal lung physiology [9, 10]. Transcriptomic analyses of various pulmonary cells, such as alveolar epithelial type II cells, alveolar macrophages, and fibroblasts, consistently reveal disruptions in lipid metabolism during fibrosis [11]. Studies have demonstrated that diminished expression of genes related to lipid, cholesterol, and steroid metabolism can reduce surfactant production in alveolar epithelial type II cells [12]. Lipid accumulation in alveolar macrophages is linked to elevated CD36 expression, leading to increased absorption of fatty acids. This imbalance in lipid metabolism can precipitate a fibrotic transformation in macrophages, culminating in augmented extracellular matrix (ECM) synthesis. Concurrently, fibroblasts display diminished activity of PPAR-γ, which can drive their metamorphosis from lipid-producing cells into myofibroblast-like entities [3]. Consequently, the disruption of lipid metabolism is recognized as a key metabolic shift in the pathogenesis of fibrosis [3, 13].

Given the strong connection between lipid metabolism disorders and IPF, there is interest in whether lipid-lowering medications have a protective impact on IPF. A cohort study within the Korean population found that the utilization of statins was linked to lower IPF risk [14]. Among individuals taking statins, the incidence rate of IPF was 15.6 cases per 100,000 person-years, which was less than the rate of 19.3 cases per 100,000 person-years observed in those not taking [14]. Another Phase III randomized clinical trial involving 624 IPF participants indicated that statins could decrease the mortality rate and the frequency of hospitalizations due to acute exacerbations [15]. Nonetheless, there is limited randomized controlled trials (RCTs), and some studies present conflicting results. An exploratory analysis of 1,450 IPF patients participating in a Phase III trial found no association between statin use and IPF progression [16]. Similarly, a review of a health management database, which included 6,665 individuals with possible or likely interstitial lung disease (ILD) and 26,660 controls, failed to find a connection between statin use and ILD development [17]. Moreover, the impact of novel lipid-lowering agents, such as PCSK9 inhibitors and NPC1L1 inhibitors, on IPF remains to be elucidated.

Mendelian randomization (MR), a recognized approach, is frequently utilized to explore the potential links between genetically influenced traits, therapeutic drug targets, and disease outcomes [18, 19]. Biological theory posits that genetic variations arise through random genetic drift and mutation, establishing a foundation for MR approach. The fundamental assumption is that these genetic variants influence phenotypic traits in a manner that is consistent and not significantly modulated by environmental influences. This concept signifies that the observed causal relationships are not confined to a specific segment of the population but are broadly applicable across the same racial or ethnic group [18]. For drug-target mendelian randomization, it employs genetic variants situated near or within proximity to the gene encoding the targeted protein as instrumental variables (IVs) to prognosticate treatment efficacy [19]. The causal inferences derived from MR are considered less prone to bias and reverse causality [20]. The evidential value of MR analysis is ranked just below that of RCTs, and it can provide significant insights that may presage the findings of RCTs [21–23].

Therefore, our study utilized the Mendelian randomization method to explore the impact of blood lipid traits on IPF risk and to assess the influence of lipid-regulatory medications on IPF.

## Methods

**Fig. 1:**
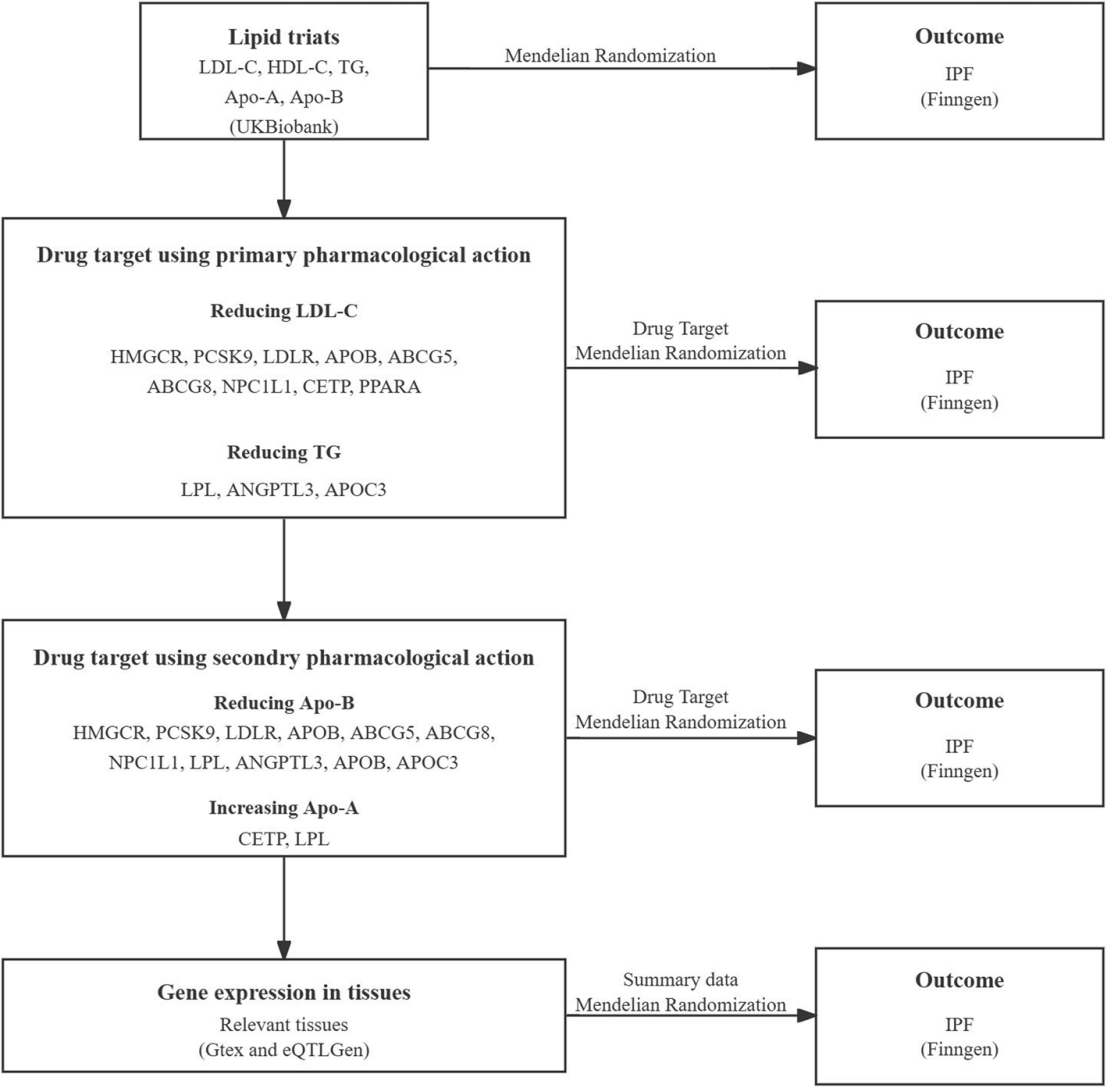
Outline of the study design

This study adhered to the Strengthening the Reporting of Observational Studies in Epidemiology-Mendelian Randomization (STROBE-GE), as detailed in Table S1 [24]. The data were derived from publicly available summary-level data from genome-wide association studies (GWAS) and expression quantitative trait loci (eQTL) studies. Comprehensive details regarding these datasets are delineated in Table S2. The schematic representation of the study design is depicted in Fig. 1. Outline of the study design.

### Genetic instrumental variables for lipid traits and lipid-modifying targets

The publicly accessible GWAS data for 3 circulating lipid traits, including high-density lipoprotein cholesterol (HDL-C, *N* = 291,830), low-density lipoprotein cholesterol (LDL-C, *N* = 318,340), and triglyceride (TG, *N* = 318,674) were obtained from the UK Biobank [25]. The UK Biobank is a vast biomedical database and research resource that includes genetic, lifestyle, and health information. It is demographically diverse, with approximately 9.2 million individuals aged 40 to 69 from across England, Wales, and Scotland invited to join the cohort, and 5.45% of them (500,000 individuals) participated in the baseline assessment [26]. Participants from the UK Biobank underwent a uniform standard lipid testing procedure, with laboratory results reported as continuous variables. The raw lipid measurements were fitted to linear regression models adjusted for covariates, including age, sex, among others [26, 27]. Genetic variants linked to these lipid traits were selected, meeting a linkage disequilibrium (LD) clumping threshold of *r*^2^ < 0.001 and a physical distance threshold of 1,000 kb.

We selected 20 prevalent lipid-lowering drugs and innovative therapeutics in accordance with recent guidelines for dyslipidemia management [28, 29]. Utilizing the DrugBank database (https://go.drugbank.com/) and pertinent scholarly articles, we undertook gene identification for the pharmacological targets of these medications [30, 31]. Comprehensive details regarding each target gene are delineated in Table S3. Based on their principal pharmacological effects, these genes are categorized into lowering LDL-C (i.e. HMGCR, PCSK9, LDLR, APOB, NPC1L1, ABCG5, ABCG8, CETP) and lowering TG (i.e. LPL, ANGPTL3, APOC3).

To simulate the lipid-lowering impact of these genes, we identified single nucleotide polymorphisms (SNPs) located within a region extending ± 100 kilobases (kb) around the gene of interest. And these SNPs should also show a significant association with lipid levels on a genome-wide scale (*p* < 5 × 10^−8^) [32]. To optimize the power of the tool, SNPs were permitted to be in weak linkage disequilibrium of less than 0.30 with one another.

To reinforce the robustness of the results, we undertook extra analyses using a novel suite of genetic tools that incorporated both Apolipoprotein A (Apo-A) and Apolipoprotein B (Apo-B). Apo-A serves as a critical transporter of HDL-C. Apo-B plays a crucial role in the formation of LDL-C and TG. Apo-A was utilized to develop tools for measuring CETP and LPL. Apo-B was employed to create genetic tools targeting HMGCR, PCSK9, LDLR, APOB, NPC1L1, ABCG5, ABCG8, LPL, ANGPTL3, and APOC3. The GWAS data for both Apo-B and Apo-A were also sourced from the UK Biobank, comprising 317,412 and 290,198 samples, respectively.

### eQTL data

We used publicly available eQTL data from the Genotype-Tissue Expression (GTEx-V8) project and eQTLGen (https://www.eqtlgen.org/). The GTEx project encompasses eQTL data across 54 distinct human tissues, with participant numbers ranging from 73 to 670 [33]. Approximately 84.6% of these samples originate from individuals of European descent. The eQTLGen Consortium has conducted cis-eQTL on up to 31,684 blood samples from 37 datasets [34]. Cis-eQTLs refer to genetic variations that have a significant link with the expression of specific genes affected by medications. These cis-eQTLs must meet the significance level of a *P*-value lower than 5 × 10^−8^ and adhere to the linkage disequilibrium criterion with an r2 value less than 0.1.

### Outcome GWAS

The GWAS data for IPF were obtained from the FinnGen Release 10 (https://r10.finngen.fi/). The FinnGen study is an extensive genomics initiative that correlates genetic variations with health data. It brings together Finnish universities, hospitals and hospital districts, the national institute for health and welfare, the Finnish biobank consortium, along with hundreds of thousands of Finns. As of December 2023, this consortium has enrolled over 412,000 participants (230,310 females and 181,871 males), analyzed more than 21.31 million genetic variants, and covered 2,408 distinct disease phenotypes. IPF cases are identified using the diagnostic code J84.1 from the International Classification of Diseases, 10th Edition (ICD-10), which included 2189 individuals with IPF and 407,609 control subjects. Of the 2189 IPF cases included, 732 were female and 1457 were male. The median age (years) at first onset of IPF was 37.54 (Females = 36.46, Males = 40.62). To affirm the efficacy of the selected genetic markers, supplementary analysis was conducted with coronary heart disease (CHD) as the benchmark outcome, serving as a positive control in our study. Summary statistics for CHD were also sourced from the FinnGen project, with 46,959 cases and 365,222 control individuals (Table S2). The GWAS data from FinnGen database utilized sex, age, genotyping batch, and ten principal components as covariates. Cases of missing data were addressed by exclusion, and all participants were of European ancestry.

### Statistical analysis

Mendelian randomization utilizes SNPs as instruments to explore the connection between exposure and outcome variables. The MR must adhere to three fundamental assumptions: (1) Correlation assumption: The IVs demonstrate a significant connection with exposure (*p* < 5 × 10^−8^). The F-statistic is also used to evaluate the hypothesis of correlation by quantifying the magnitude of each genetic variant. A larger F statistic (> 10) suggests a little chance of weak instrumental variable bias [21, 22]; (2) Independence assumption: The IVs should be unconfounded, meaning they are unrelated to factors that could affect both exposure and outcome, ensuring that the observed associations are uniquely due to the exposure under investigation; (3) Exclusivity assumption: The IVs do not have a direct correlation with the outcome, nor any other means apart from exposure to correlate with the outcome [35].

The principal MR analysis was executed utilizing the inverse variance weighted (IVW) method, which has been shown to have the most pronounced statistical impact. Following the statistical methods similar to previous studies, three additional MR methods (MR-Egger, weighted median, and weighted mode) were also implemented as complementary approaches [36–39]. The MR-Egger regression is a method that accounts for potential pleiotropy by including an intercept in the regression model. The assumptions checked in this method include linearity, homogeneity and directional pleiotropy. It allows for the possibility of directional pleiotropy and aims to control for this by including an intercept term in the regression model [37]. The weighted median method offers robustness against violations of the pleiotropy-free assumption, as long as pleiotropic variants constitute a minor fraction of the instruments [38]. This method operates under the assumption of equal effect sizes, positing that all valid genetic instruments impart a uniform influence on the outcome, thus representing a stronger assumption compared to that of the MR-Egger regression. In contrast, the weighted mode method allocates greater influence to the most prevalent genetic instrument while still maintaining the assumption of majority valid instruments—that the majority of genetic instruments are valid and only a minority are invalid. It also assumes equal effect sizes, suggesting that all valid instruments have an identical impact on the outcome. To improve the strength of the results, the contamination mixture method (ConMix) and robust adjusted profile score (RAPS) were also utilized. Compared with other methods, ConMix had the lowest mean squared error [40]. MR-RAPS considers special pleiotropicity and can provide reliable inferences for MR analysis utilizing weak instrumental variables [41]. Considering our repetitive calculations, we applied Benjamini–Hochberg false-discovery rate (FDR) procedure to adjust the raw *p*-values [42]. The results of all estimates are typically presented as odds ratio (OR) along with its 95% confidence interval (CI).

In this study, we initially utilized two-sample MR analysis to explore the causal effect between lipids and IPF risk, after harmonizing the alleles for consistency. Subsequently, we employed drug-targeted mendelian randomization to ascertain whether a relationship exists between genetically proxied lipid-modifying interventions and IPF. For drug targets showing suggestive significance, we carried out a summary-data-based MR (SMR) analysis to explore the association between gene expression and IPF, synthesizing data from GWAS and eQTL studies.

To reinforce the validity of the MR model's assumptions and support the reliability of findings, we undertook a series of extra sensitivity analyses. Methods included MR-Pleiotropy Residual Sum and Outlier (MR-PRESSO) for horizontal pleiotropy; MR-Egger intercept tests for directional pleiotropy [37, 43]; Cochran’s Q test for heterogeneity [44]; and leave-one-out MR analyses to evaluate whether a single SNP has an excessive impact on MR analysis [45]. Scatter plot was also used for visual inspection of outliers in SNP-specific causal estimates. Then, we used the online tool mRnd (http://cnsgenomics.com/shiny/mRnd/) to calculate statistical power [46]. Input parameters for power calculations in this tool include sample size, type-I error rate, proportion of cases in the study, odds ratio, and proportion of variance explained for the association between the SNP. A statistical power greater than 0.8 typically indicates that a study has a high detection capability, effectively countering the impact of sampling error and random variation, and reducing the risk of Type II errors (i.e., false negatives) [46]. In the context of the SMR method, we applied the heterogeneity in dependent instruments (HEIDI) test to appraise the robustness and reliability of the results. A *P*-value cutoff of less than 0.05 was set to suggest that the observed association may be a result of linkage disequilibrium.

The statistical procedures outlined above were conducted using the R programming language (version 4.3.0), with the aid of the packages “TwoSampleMR” and “MR-PRESSO”. Findings at a *P*-value threshold below 0.05 were deemed statistically meaningful.

## Results

### Lipid traits and IPF risk

**Fig. 2:**
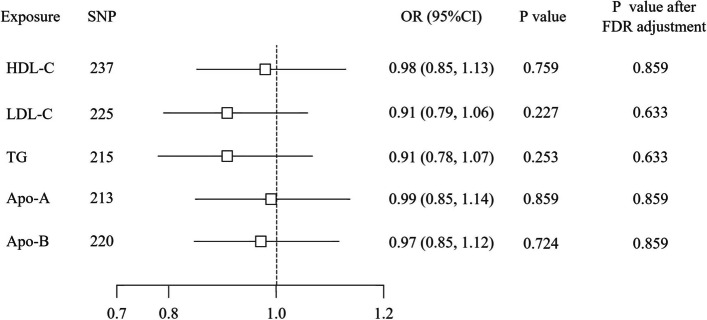
Forest plots of the association between blood lipid traits and IPF

In the MR study, 237 SNPs were selected for HDL-C, 225 SNPs for LDL-C, 215 SNPs for TG, 213 SNPs for Apo-A, and 220 SNPs for Apo-B as IVs (Tables S4-8). The IVW method indicated that genetically predicted HDL-C with an OR of 0.978 and 95%CI from 0.849 to 1.127 (*P* = 0.761), LDL-C with an OR of 0.927 and 95% CI from 0.801 to 1.071 (*P* = 0.302), TG with an OR of 0.908 and 95% CI from 0.777 to 1.060 (*P* = 0.221), Apo-A with an OR of 0.993 and 95% CI from 0.860 to 1.147 (*P* = 0.924), and Apo-B with an OR of 0.990 and 95% CI from 0.856 to 1.145 (*P* = 0.895) were not related to IPF risk (Fig. 2. Forest plots of the association between blood lipid traits and IPF. Five additional MR methods also yielded consistent results (Table S9). Fig. S1 displayed scatter plots showing the assocaition between lipid traits and IPF. Each SNP had an F-statistics value above the threshold of 10, as detailed in Table S4-8. The heterogeneities for HDL-C (P_MR-Egger_ = 0.017, P_IVW_ = 0.017) and Apo-B (P_MR-Egger_ = 0.033, P_IVW_ = 0.025) were detected (Table S10). No directional pleiotropies were found in sensitivity analyses.

### Lipid-lowering drugs and IPF risk

We selected SNPs that predict the lipid-modifying effect of genes responsible for the targets affected by lipid-lowering medications as IVs. A total of 21 SNPs as IVs in HMGCR, 5 SNPs in NPC1L1, 33 SNPs in PCSK9, 30 SNPs in APOB, 22 SNPs in ABCG5, 23 SNPs in ABCG8, 42 SNPs in LDLR, 11 SNPs in CETP, 23 SNPs in ANGPTL3, 31 SNPs in APOC3, and 49 SNPs in LPL were identified (Table S11). The positive control assessment revealed a substantial association between drug target inhibitors and lower CHD risk, indicating the efficacy of genetic tools, except for APOB inhibitors (Fig. S2, Table S12). Scatter plots are shown in Fig. S3.

**Fig. 3:**
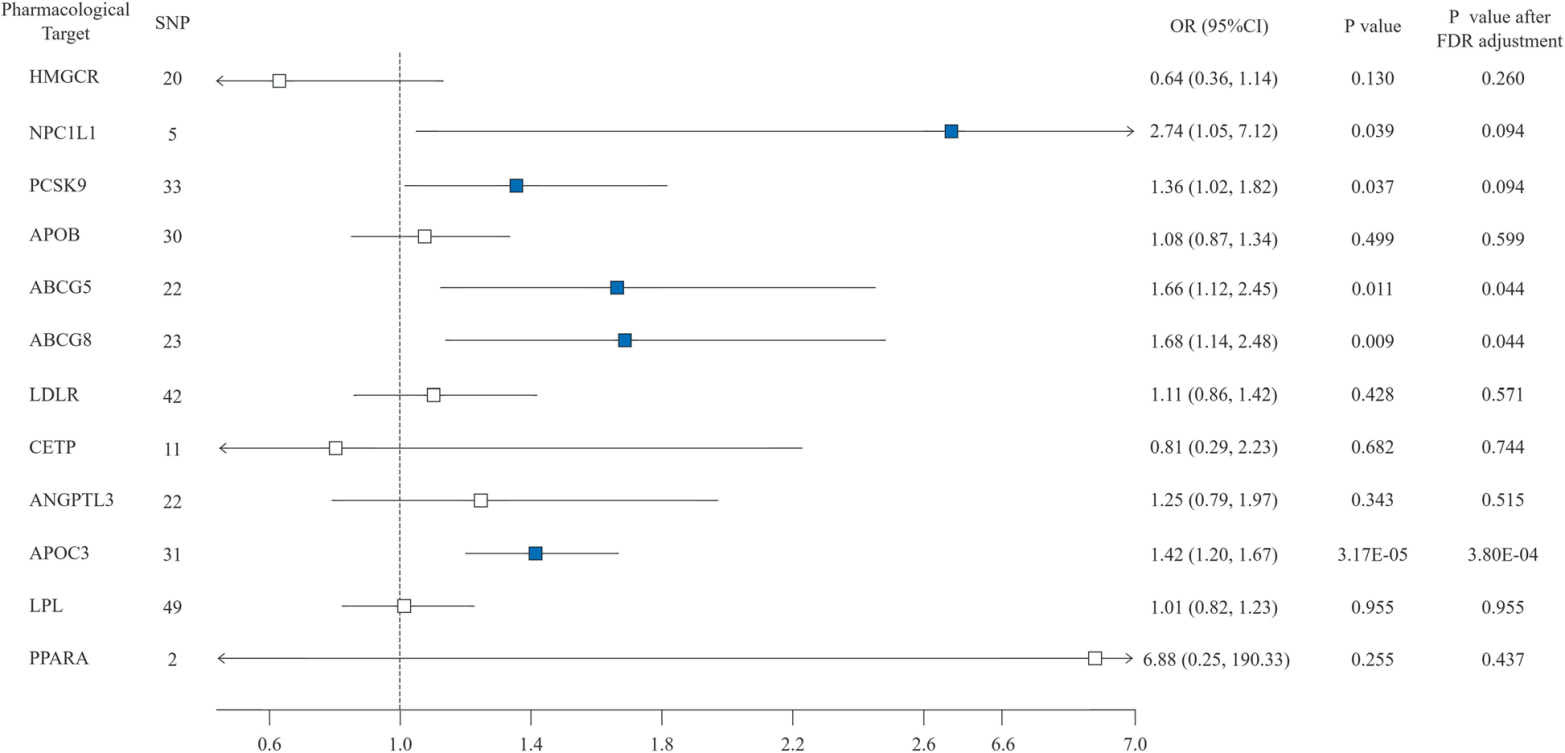
Forest plots of the association between genetically proxied lipid-modifying drug and IPF using primary effect

The association between genetic metabolites influenced by 12 lipid-modifying drugs and IPF is shown in Fig. 3. Forest plots of the association between genetically proxied lipid-modifying drug and IPF using primary effect. The IVW-MR analysis showed that the reduced LDL-C level by inhibitors or enhancements of NPC1L1, PCSK9, ABCG5, and ABCG8 increased IPF risk (OR = 2.74, 95% CI: 1.05 – 7.12, *P* = 0.039; OR = 1.36, 95% CI: 1.02 – 1.82, *P* = 0.037; OR = 1.66, 95% CI: 1.12 – 2.48, *P* = 0.011; OR = 1.68, 95% CI: 1.14 – 2.48, *P* = 0.009). Inhibition of APOC3, similar to the decrease in Apo-B level, was significantly correlated with an increased IPF risk (OR = 1.42, 95% CI: 1.20 – 1.67, *P* = 3.17 × 10^–5^). The result using other five supplementary methods remained directionally concordant with IVW. Conversely, the MR analyses did not reveal any causal influence on the risk of IPF from genetic mimicries of HMGCR, LDLR, APOB, LPL, ANGPTL3, CETP, and PPARA inhibitors (all *P* > 0.05) (Fig. 3. Forest plots of the association between genetically proxied lipid-modifying drug and IPF using primary effect, Table S13). After FDR correction, ABCG5, ABCG8 and APOC3 were found to be signifcantly associated with IPF risk (P_FDR_ < 0.05, Fig. 3), NPC1L1 and PCSK9 had a suggestive effect on IPF risk. The F statistics of each genetic tool range from 29.94 to 3712.59 (Table S11). Scatter plots of the association of lipid-modifying gene targets with IPF risk were presented in Fig. S4. Drug targets such as NPC1L1, ABCG5, ABCG8, APOC3, and PPARA exhibit high statistical power, with values ranging from 0.88 to 1.00, which provides robustness to our findings. However, targets like APOB, CETP, and LPL show lower statistical power, underscoring the need for a cautious interpretation of the associated results. Refer to Table S14 for details regarding the statistical power associated with the MR analyses.

**Fig. 4:**
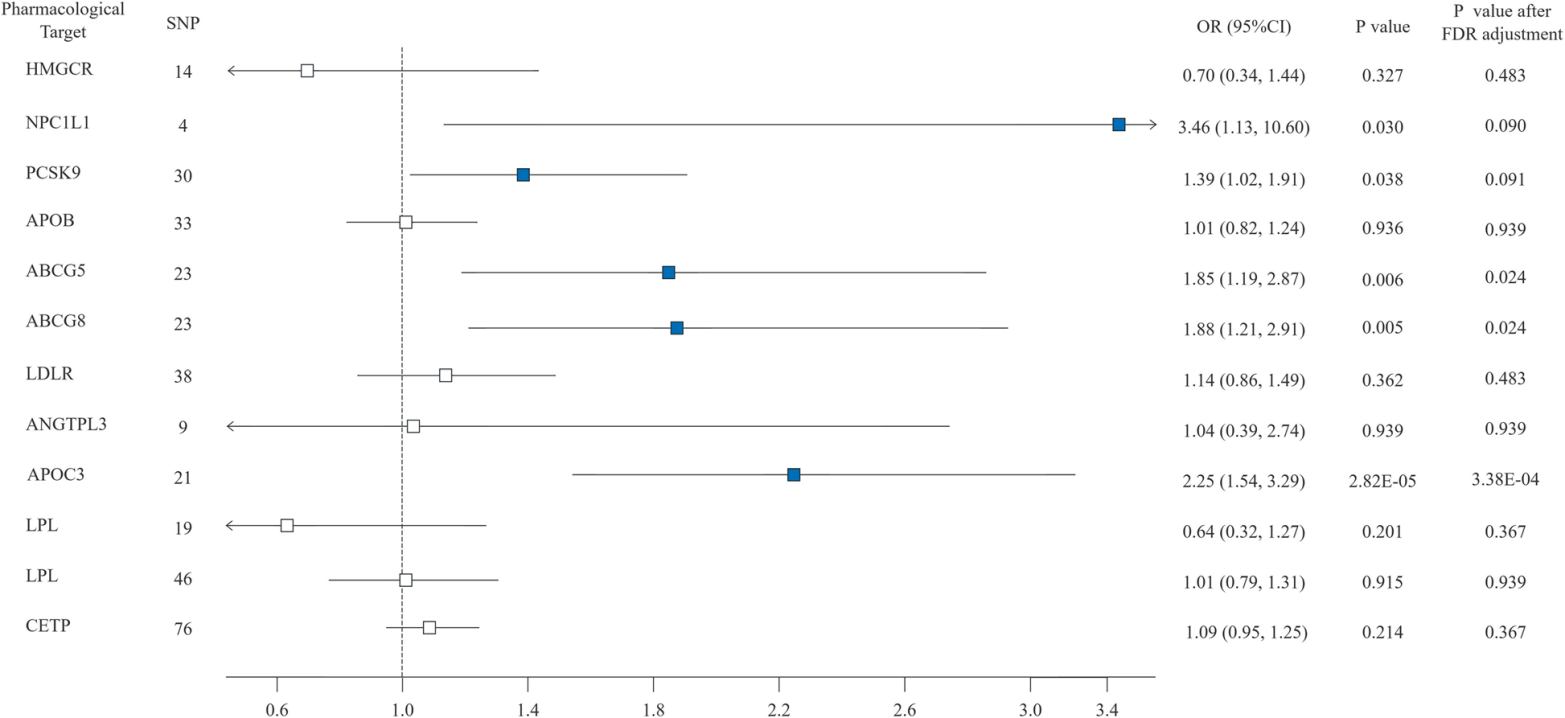
Forest plots of the association between genetically proxied lipid-modifying drug and IPF using alternative effect

Similar results were also obtained from the genetic mimicry analysis with secondary effects on Apo-B and Apo-A. The decreased Apo-B level mediated by NPC1L1, PCSK9, ABCG5, ABCG8, and APOC3 gene targeted drugs were respectively associated with a higher IPF risk (OR = 3.46, 95% CI: 1.13 – 10.60, *P* = 0.030; OR = 1.39, 95% CI: 1.02 – 1.91, *P* = 0.038; OR = 1.85, 95% CI: 1.19 – 2.87, *P* = 0.006; OR = 1.88, 95% CI: 1.21 – 2.91, *P* = 0.005; OR = 2.25, 95% CI: 1.54 – 3.29, *P* = 2.82 × 10^–5^) (Fig. 4. Forest plots of the association between genetically proxied lipid-modifying drug and IPF using alternative effect). After FDR correction, ABCG5, ABCG8 and APOC3 were found to be signifcantly associated with IPF risk (P_FDR_ < 0.05). Other genetic mimicries of drug targets showed no significant association with IPF (Table S15). The F statistics values for each SNP exceeded 30 (Table S16). Drug targets like NPC1L1, ABCG5, ABCG8, APOC3 showed high statistical power (> 0.8), while APOB, ANGPTL3, and LPL show lower statistical power (Table S17).

The MR-Egger intercept examination did not uncover any indications of pleiotropy, which enhances the credibility of causal inferences (Tables S18-S19). Leave-one-out analyses showed that the IVW method consistently produced results in line with the overall estimate even after excluding each SNP sequentially (Figs. S5-S6). Further analyses were carried out using stricter LD thresholds (*r*2 < 0.2, *r*2 < 0.1, *r*2 < 0.01, and *r*2 < 0.001) for these genes. These analyses did not significantly change the direction of the beta values, although the statistical power was reduced by excluding multiple SNPs (Table S20).

### Gene expression and IPF risk

Given that the NPC1L1, PCSK9, ABCG5, ABCG8, and APOC3 genes showed an association with IPF, genetic variants linked to these gene expressions in blood and relevant tissues were used as IVs for additional validation. However, the limited sample size of eQTL data prevented us from identifying eligible cis-eQTLs for APOC3 in related tissues.

SMR analysis results suggested that a higher expression level of PCSK9 in whole blood was associated with a lower risk of IPF (OR = 0.71, 95% CI: 0.50 – 0.99, *P* = 0.043) (Table S20). SMR analysis also found that there was a tendency towards significance in the connection between high expression of the NPC1L1 gene in adipose subcutaneous and lower IPF risk (OR = 0.85, 95% CI: 0.73 – 1.00, *P* = 0.051) (Table S20). No significant association was detected between the ABCG5 gene expression levels in the spleen (OR = 1.11, 95% CI: 0.99 – 1.24, *P* = 0.087), ABCG8 expression in colon transverse (OR = 1.04, 95% CI: 0.86 – 1.25, *P* = 0.713) and IPF risk. The HEIDI test results demonstrated that the observed associations were unlikely to be due to linkage disequilibrium (*p* > 0.05) (Table S21).

## Discussion

To our best knowledge, this study represents the inaugural application of MR analysis to explore the relationship between lipids, lipid-modifying pharmacological interventions, and IPF. Our results offer genetic corroboration for the proposition that PCSK9 inhibitors may elevate the risk of IPF. Notably, no evidence was found to suggest that lipid traits have a causal association with IPF risk, which implies that the mechanism by which PCSK9 inhibitors influence IPF risk may be distinct from its effects on lipid metabolism. Additionally, the study uncovered preliminary evidence hinting at a potential positive association between NPC1L1 inhibition and a higher IPF risk. The discovery of these results illuminates the possible adverse effects linked to the use of lipid-modifying drugs and offers an understanding of potential risk factors that may be investigated for IPF management.

Two previous MR studies have explored the association between blood lipid traits and IPF [47, 48]. An MR analysis led by Sizheng Steven Zhao presented a seemingly paradoxical finding: a higher LDL levels and statins-use were associated with reduced IPF risk [47]. In contrast, the MR analysis by Yan Jiang found that there is a causual effect between Apo-B and IPF risk [48]. We argue that the reasons our MR findings differ from those in previous studies are as follows. Firstly, the impact of sample size and sample overlap cannot be ignored. These two MR analyses included a larger scale of blood lipid trait data and both used the same IPF GWAS data as outcome. This IPF GWAS data pooled the meta-analysis results of 4,125 IPF cases and 20,464 control individuals [48]. The IPF meta-analysis data came from five cohort studies, including two studies that specifically included IPF cases from the UK population [48]. However, in Sizheng Steven Zhao's MR analysis, the data on statins-use came from the UK Biobank, and in Yan Jiang's study, the blood lipid trait data also came from the UK Biobank. Neither of these two MR studies assessed the degree of overlap between the exposure and outcome samples and its potential impact on the study results. Secondly, the impact of horizontal pleiotropy and confounding factors may have caused bias in causal inference. In the sensitivity analysis of the relationship between LDL and IPF, Cochran's Q test suggested the possible existence of horizontal pleiotropy (Q *p*-value = 6.13E-05), which may distort the inference of the causal relationship [47]. In addition, in Sizheng Steven Zhao's study, it was also found that the BMI is a risk factor for IPF. Given the close connection that may exist between statins-use, LDL levels, and BMI, the specific impact of BMI as a confounding factor and its interpretation are still unclear [47]. Lastly, the data on the use of statins depends on self-reports, which may introduce recall bias and reporting bias, thereby affecting the accuracy of causal inference.

Although the current evidence did not support serum lipid traits as causal risk factors for IPF, the result should be interpreted with caution. Firstly, lipids are categorized into four primary groups, including glycerides, fatty acids, non-glycerides, and lipoproteins. The plasma lipid traits predominantly featured in this MR study pertain to the lipoprotein class. However, extant literature suggests that dysregulation of phospholipid and sphingolipid metabolism is a more substantial contributor to IPF pathophysiology [9, 49, 50]. A lipidomics analysis has revealed that alterations in the plasma lipid profile of IPF patients are predominantly within the glycerophospholipid class. Among the 159 glycerolipids examined, 30 exhibited significant disparities between the IPF group and controls [50]. Moreover, the lung, being a lipid-rich organ, engages in intricate lipid metabolic processes. Compared with blood lipids, alveolar lipid levels may provide a more precise reflection of metabolic disturbances. Despite a paucity of research directly comparing lipidomics profiles between bronchoalveolar lavage fluid and blood samples from IPF individuals, the work of Marissa O 'Callaghan et al. offers some valuable insights. This study observed a marked increase in total lipid content within the lung tissue of IPF individuals relative to controls [51]. In addition, they also assessed pulmonary fat attenuation volume through chest CT images (CT_PFAV_). The median CT_PFAV_ in IPF was greater compared to controls, however, there was no association observed with serum lipids and body mass index [51]. These findings imply that extracellular lipids within the lung may have a closer relationship with IPF than blood lipid traits. Future MR studies could explore the bidirectional causality between intrapulmonary lipid traits and IPF.

The MR study we conducted revealed a suggestive association between PCSK9 inhibitors and IPF. Contrary to manifesting as a protective effect, this association was characterized as a risk factor, which contradicts findings from previous studies. PCSK9, an enzyme, is essential for regulating cholesterol metabolism and maintaining cardiovascular health. Recent studies have shown that the PCSK9 gene may be implicated in the fibrotic processes of the liver, heart, kidney, and other organs. A study by Stefania Grimaudo revealed that increasing PCSK9 expression in male mice led to faster progression of liver fibrosis [52]. This research also described a protective role for the PCSK9 loss-of-function mutation against the progression of liver fibrosis. Subsequent research has indicated that anti-PCSK9 treatment may hold the potential to mitigate liver fibrosis by modulating the AMPK/mTOR/ULK1 signaling pathway, thus reducing hypoxia-induced autophagy in hepatocytes [53]. Another in vitro experiment showed that increasing PCSK9 levels could enhance the transition of cardiac fibroblasts into myofibroblasts, impacting fibrosis post-myocardial infarction [54]. Similar protective effects were also observed in renal fibrosis. Danyu Wu et al. developed a therapeutic vaccine targeting PCSK9. The results indicated that this vaccine could ameliorate kidney fibrosis by controlling fatty acid β-oxidation [55].

We postulate that several reasons may account for the inconsistent results observed. Firstly, the pathogenesis of IPF is highly complex, and the role of PCSK9 is also not singular. Previous research has concentrated on the anti-fibrotic effect of PCSK9 inhibitors in different organs through autophagy and oxidative regulation, rather than its impact on lipid levels. The balance between the anti-fibrotic effects of PCSK9 inhibitors through similar pathways or the pro-fibrotic effect through other pathways in lung tissue requires further investigation. Secondly, the expression levels of target genes can vary in different tissues, as evidenced by our SMR analysis of PCSK9 gene expression across different tissues. This variability can influence the therapeutic efficacy of drugs in a tissue-specific manner. Thirdly, drug-target Mendelian randomization analysis primarily models impact of gene inhibitors or blockers on outcomes using SNPs within the gene-specific action range (± 100 kb). It provides information on the trends of connections rather than the tangible therapeutic advantages of drugs in practical situations. Numerous factors, such as drug dosage, when the drug is given, how inter-individuals metabolize drugs, and how well drugs attach to their intended targets, need to be further considered. Consequently, further RCTs are essential to confirm these observational findings.

Our findings also imply that there is a potential for increased IPF risk with exposure to NPC1L1 inhibitors, although the SMR analysis only suggested a near-positive result. Ezetimibe is known as the primary drug for NPC1L1 inhibitors, which are responsible for facilitating the absorption of dietary cholesterol by NPC1L1 protein [29]. To date, limited research has examined the influence of ezetimibe on the onset of IPF. Chanho Lee et al. conducted a retrospective study of the medical records across three different hospitals and discovered that individuals with IPF who consistently used ezetimibe had lower all-cause mortality and lung function decline rates [56]. They also found that ezetimibe could prevent mice from developing bleomycin-induced pulmonary fibrosis by suppressing mTORC1 activity in vitro study. The enhancement of autophagy in mouse lung fibroblasts mediates the anti-fibrotic effect of ezetimibe, rather than through lipid-lowering properties [56]. There may exist differences in the pathogenesis between drug-induced pulmonary fibrosis and IPF, which could account for the discrepancies between our MR findings and the in vitro results.

This MR research also has some limitations. Firstly, despite the numerous sensitivity analyses conducted that have reinforced the reliability of our results, the possibility of horizontal pleiotropy cannot be entirely dismissed. Although the original *P*-value may suggest an association between NPC1L1 and PCSK9 with IPF, the adjusted *P*-value indicates that this association may not be significant enough to rule out chance. Thus, the results should be interpreted with caution. Further research is needed in the future to validate the original findings, or a larger sample size may be required to provide sufficient statistical power to overcome the impact of multiple comparisons. Secondly, the sample size of eQTL data is limited, and there are no eligible eQTLs for NPC1L1, ABCG5, ABCG8, and APOC3 in hepatic and pulmonary tissues, which are the primary organs involved in lipid metabolism. This limitation may result in an underestimation of the role these genes play in the pathogenesis of IPF. Thirdly, the initial GWAS data did not categorize by specific subtypes (such as the extent of LDL-C elevation). Consequently, this study was constrained from performing a stratified analysis. Furthermore, the reliance on diagnostic codes to define IPF within the FinnGen study may not fully encapsulate the clinical and pathological nuances of the disease. This approach could inadvertently include cases that do not actually represent IPF, potentially skewing the analysis. Consequently, the estimated strength of association between IPF and specific genetic variants might be subject to overestimation or underestimation. An approach that should be contemplated with the availability of more specific datasets in the future. Lastly, the IPF GWAS data was obtained from an isolated European population. We lack a validation cohort because other GWAS data for IPF may have sample overlap with lipid exposure. When extrapolating these results to different ethnic groups, caution should be exercised. Additional research encompassing a diverse array of populations is necessary.

## Conclusions

In summary, this study does not support blood lipid traits (i.e., TG, LDL-C, HDL-C, Apo-A, and Apo-B) as a direct risk factor for IPF and should be interpreted with caution. An increased expression level of the PCSK9 gene was found to correlate with a lower risk of IPF. Conversely, the use of PCSK9 inhibitors was associated with an elevated risk of IPF. Further studies are essential to gain a more comprehensive understanding of the underlying mechanisms and to assess the possible effect of PCSK9 inhibitors in IPF progression through a series of preclinical and clinical trials.

### Supplementary Information

Additional file 1: Table S1. STROBE-MR checklist. Table S2. Phenotype descriptions and distributions. Table S3. Lipid-lowering drug classes, substances, and target genes. Table S4. Genetic variants that were used as instrumental variables for high-density lipoprotein cholesterol. Table S5. Genetic variants that were used as instrumental variables for low-density lipoprotein cholesterol. Table S6. Genetic variants that were used as instrumental variables for triglyceride. Table S7. Genetic variants that were used as instrumental variables for apolipoprotein A. Table S8. Genetic variants that were used as instrumental variables for apolipoprotein B. Table S9. Association of genetically proxied lipid traits with risk of IPF. Table S10. Heterogeneity and pleiotropy tests of instrument effects （lipid traits on IPF). Table S11. Characteristics of lipid-modifying genetics variants in target genes using primary effect. Table S12. Association of genetically proxied lipid-modifying drugs with risk of CHD. Table S13. Association of genetically proxied lipid-modifying drugs with risk of IPF using primary effect. Table S14. Statistical power estimates for drug-target MR analyses using primary effect. Table S15. Association of genetically proxied lipid-modifying drugs with risk of IPF using alternative effect. Table S16. Characteristics of lipid-modifying genetics variants in target genes using alternative effect. Table S17. Statistical power estimates for drug-target MR analyses using alternative effect. Table S18. Heterogeneity and pleiotropy tests of instrument effects (primary lipid-modifying effect). Table S19. Heterogeneity and pleiotropy tests of instrument effects (alternative lipid-modifying effect). Table S20. Association of genetic mimicry of lipid-modifying drugs with risk of IPF accounting for LD structure. Table S21. Association between gene expression in tissues of identified targets and IPF in the SMR analysis.

Additional file 2: Fig. S1. Scatter plots of the association between lipid traits and IPF; **A.** High-density lipoprotein cholesterol on idiopathic pulmonary fibrosis; **B.** Low-density lipoprotein cholesterol on idiopathic pulmonary fibrosis; **C.** Triglyceride on idiopathic pulmonary fibrosis; **D.** Apolipoprotein A on idiopathic pulmonary fibrosis; **E.** Apolipoprotein B on idiopathic pulmonary fibrosis.

Additional file 3: Fig. S2. Forest plots of the association between genetically proxied lipid-modifying drug and CHD risk.

Additional file 4: Fig. S3. Scatter plots of the association between genetically proxied lipid-modifying gene targets and CHD. **A.** HMGCR on coronary heart disease; **B.** NPC1L1 on coronary heart disease; **C.** PCSK9 on coronary heart disease; **D.** APOC on coronary heart disease; **E.** ABCG5 on coronary heart disease; **F.** ABCG8 on coronary heart disease; **G.** LDLR on coronary heart disease; **H.** CETP on coronary heart disease; **I.** ANGPTL3 on coronary heart disease; **J.** APOC3 on coronary heart disease; **K.** LPL on coronary heart disease; **L.** PPARA on coronary heart disease.

Additional file 5: Fig. S4. Scatter plots of the association between genetically proxied lipid-modifying gene targets and IPF. **A.** HMGCR on idiopathic pulmonary fibrosis; **B.** NPC1L1 on idiopathic pulmonary fibrosis; **C.** PCSK9 on idiopathic pulmonary fibrosis; **D.** APOC on idiopathic pulmonary fibrosis; **E.** ABCG5 on idiopathic pulmonary fibrosis; **F.** ABCG8 on idiopathic pulmonary fibrosis; **G.** LDLR on idiopathic pulmonary fibrosis; **H.** CETP on idiopathic pulmonary fibrosis; **I.** ANGPTL3 on idiopathic pulmonary fibrosis; **J.** APOC3 on idiopathic pulmonary fibrosis; **K.** LPL on idiopathic pulmonary fibrosis; **L.** PPARA on idiopathic pulmonary fibrosis.

Additional file 6: Fig. S5. Plots of “leave-one-out” analyses for MR analyses of the causal effect of lipid-modifying drugs on IPF using primary effect. **A.** Genetic mimicries of NPC1L1 inhibitor on idiopathic pulmonary fibrosis; **B.** Genetic mimicries of PCSK9 inhibitor on idiopathic pulmonary fibrosis; **C.** Genetic mimicries of ABCG5 enhancement on idiopathic pulmonary fibrosis; **D.** Genetic mimicries of ABCG8 enhancement on idiopathic pulmonary fibrosis; **E.** Genetic mimicries of APOC3 blocker on idiopathic pulmonary fibrosis.

Additional file 7: Fig. S6. Plots of “leave-one-out” analyses for MR analyses of the causal effect of lipid-modifying drugs on IPF using alternative effect. **A.** Genetic mimicries of NPC1L1 inhibitor on idiopathic pulmonary fibrosis; **B.** Genetic mimicries of PCSK9 inhibitor on idiopathic pulmonary fibrosis; **C.** Genetic mimicries of ABCG5 enhancement on idiopathic pulmonary fibrosis; **D.** Genetic mimicries of ABCG8 enhancement on idiopathic pulmonary fibrosis; **E.** Genetic mimicries of APOC3 blocker on idiopathic pulmonary fibrosis.

## Data Availability

No datasets were generated or analysed during the current study.

## Abbreviations

IPF: Idiopathic pulmonary fibrosis
MR: Mendelian randomization
SMR: Summary-data-based MR
OR: Odds ratio
95%CI: 95% Confidence interval
RCTs: Randomized controlled trials
ILD: Interstitial lung disease
GWAS: Genome-wide association studies
eQTL: Expression quantitative trait loci
HDL-C: High-density lipoprotein cholesterol
LDL-C: Low-density lipoprotein cholesterol
TG: Triglyceride
Kb: Kilobases
Apo-A: Apolipoprotein A
Apo-B: Apolipoprotein B
LD: Linkage disequilibrium
SNP: Single nucleotide polymorphism
GTEx-V8: Genotype-Tissue Expression project
CHD: Coronary heart disease
IVW: Inverse-variance-weighted
IVs: Instrumental variables
ConMix: Contamination mixture method
RAPS: Robust adjusted profile score
MR-PRESSO: MR-Pleiotropy Residual Sum and Outlier
HEIDI: Heterogeneity in dependent instruments
CT_PFAV_: Pulmonary fat attenuation volume through chest CT images

## References

[1] Moss BJ, Ryter SW, Rosas IO. Pathogenic mechanisms underlying Iidiopathic pulmonary fibrosis. Annu Rev Pathol. 2022;17:515–46. 34813355 10.1146/annurev-pathol-042320-030240

[2] Hamanaka RB, Mutlu GM. Metabolic requirements of pulmonary fibrosis: role of fibroblast metabolism. FEBS J. 2021;288:6331–52. 33393204 10.1111/febs.15693 PMC8253875

[3] Rajesh R, Atallah R, Bärnthaler T. Dysregulation of metabolic pathways in pulmonary fibrosis. Pharmacol Ther. 2023;246: 108436. 37150402 10.1016/j.pharmthera.2023.108436

[4] Justet A, Klay D, Porcher R, Cottin V, Ahmad K, Molina Molina M, et al. Safety and efficacy of pirfenidone and nintedanib in patients with idiopathic pulmonary fibrosis and carrying a telomere-related gene mutation. Eur Respir J. 2021;57:2003198. 33214205 10.1183/13993003.03198-2020

[5] Behr J, Nathan SD, Wuyts WA, Mogulkoc Bishop N, Bouros DE, Antoniou K, et al. Efficacy and safety of sildenafil added to pirfenidone in patients with advanced idiopathic pulmonary fibrosis and risk of pulmonary hypertension: a double-blind, randomised, placebo-controlled, phase 2b trial. Lancet Respir Med. 2021;9:85–95. 32822614 10.1016/S2213-2600(20)30356-8

[6] Lederer DJ, Martinez FJ. Idiopathic pulmonary fibrosis. N Engl J Med. 2018;378:1811–23. 29742380 10.1056/NEJMra1705751

[7] Saito S, Alkhatib A, Kolls JK, Kondoh Y, Lasky JA. Pharmacotherapy and adjunctive treatment for idiopathic pulmonary fibrosis (IPF). J Thorac Dis. 2019;11:S1740–54. 31632751 10.21037/jtd.2019.04.62 PMC6783717

[8] Zhao X, Kwan JYY, Yip K, Liu PP, Liu F-F. Targeting metabolic dysregulation for fibrosis therapy. Nat Rev Drug Discov. 2020;19:57–75. 31548636 10.1038/s41573-019-0040-5

[9] Burgy O, Loriod S, Beltramo G, Bonniaud P. Extracellular lipids in the lung and their role in pulmonary fibrosis. Cells. 2022;11:1209. 35406772 10.3390/cells11071209 PMC8997955

[10] Tian Y, Duan C, Feng J, Liao J, Yang Y, Sun W. Roles of lipid metabolism and its regulatory mechanism in idiopathic pulmonary fibrosis: a review. Int J Biochem Cell Biol. 2023;155:106361. 36592687 10.1016/j.biocel.2022.106361

[11] Xu Y, Mizuno T, Sridharan A, Du Y, Guo M, Tang J, et al. Single-cell RNA sequencing identifies diverse roles of epithelial cells in idiopathic pulmonary fibrosis. JCI Insight. 2016;1:e90558. 27942595 10.1172/jci.insight.90558 PMC5135277

[12] Reyfman PA, Walter JM, Joshi N, Anekalla KR, McQuattie-Pimentel AC, Chiu S, et al. Single-cell transcriptomic analysis of human lung provides insights into the pathobiology of pulmonary fibrosis. Am J Respir Crit Care Med. 2019;199:1517–36. 30554520 10.1164/rccm.201712-2410OC PMC6580683

[13] Chen R, Dai J. Lipid metabolism in idiopathic pulmonary fibrosis: from pathogenesis to therapy. J Mol Med (Berl). 2023;101:905–15. 37289208 10.1007/s00109-023-02336-1

[14] Jang HJ, Lee DY, Loloci G, Jeong J, Choi W-I. Association between the use of statins and risk of interstitial lung disease/idiopathic pulmonary fibrosis: time-dependent analysis of population-based nationwide data. Eur Respir J. 2023;62:2300291. 37202155 10.1183/13993003.00291-2023

[15] Kreuter M, Bonella F, Maher TM, Costabel U, Spagnolo P, Weycker D, et al. Effect of statins on disease-related outcomes in patients with idiopathic pulmonary fibrosis. Thorax. 2017;72:148–53. 27708114 10.1136/thoraxjnl-2016-208819 PMC5284334

[16] Kreuter M, Lederer DJ, Cottin V, Kahn N, Ley B, Vancheri C, et al. Concomitant medications and clinical outcomes in idiopathic pulmonary fibrosis. Eur Respir J. 2019;54:1901188. 31537696 10.1183/13993003.01188-2019 PMC6906546

[17] Saad N, Camus P, Suissa S, Ernst P. Statins and the risk of interstitial lung disease: a cohort study. Thorax. 2013;68:361–4. 23299962 10.1136/thoraxjnl-2012-201823

[18] Smith GD. Mendelian randomization for strengthening causal inference in observational studies: application to gene × environment interactions. Perspect Psychol Sci. 2010;5:527–45. 26162196 10.1177/1745691610383505

[19] Schmidt AF, Finan C, Gordillo-Marañón M, Asselbergs FW, Freitag DF, Patel RS, et al. Genetic drug target validation using Mendelian randomisation. Nat Commun. 2020;11:3255. 32591531 10.1038/s41467-020-16969-0 PMC7320010

[20] Smith GD, Ebrahim S. “Mendelian randomization”: can genetic epidemiology contribute to understanding environmental determinants of disease? Int J Epidemiol. 2003;32:1–22. 12689998 10.1093/ije/dyg070

[21] Davies NM, Holmes MV, Davey SG. Reading Mendelian randomisation studies: a guide, glossary, and checklist for clinicians. BMJ. 2018;362:k601. 30002074 10.1136/bmj.k601 PMC6041728

[22] Williams DM, Finan C, Schmidt AF, Burgess S, Hingorani AD. Lipid lowering and alzheimer disease risk: a mendelian randomization study. Ann Neurol. 2020;87:30–9. 31714636 10.1002/ana.25642 PMC6944510

[23] Reay WR, Cairns MJ. Advancing the use of genome-wide association studies for drug repurposing. Nat Rev Genet. 2021;22:658–71. 34302145 10.1038/s41576-021-00387-z

[24] Skrivankova VW, Richmond RC, Woolf BAR, Yarmolinsky J, Davies NM, Swanson SA, et al. Strengthening the reporting of observational studies in epidemiology using Mendelian randomization: The STROBE-MR statement. JAMA. 2021;326:1614–21. 34698778 10.1001/jama.2021.18236

[25] Sinnott-Armstrong N, Tanigawa Y, Amar D, Mars N, Benner C, Aguirre M, et al. Genetics of 35 blood and urine biomarkers in the UK Biobank. Nat Genet. 2021;53:185–94. 33462484 10.1038/s41588-020-00757-z PMC7867639

[26] Fry A, Littlejohns TJ, Sudlow C, Doherty N, Adamska L, Sprosen T, et al. Comparison of sociodemographic and health-related characteristics of UK biobank participants with those of the general population. Am J Epidemiol. 2017;186:1026–34. 28641372 10.1093/aje/kwx246 PMC5860371

[27] Fry A, Littlejohns TJ, Sudlow C, Doherty N, Allen NE. OP41 The representativeness of the UK Biobank cohort on a range of sociodemographic, physical, lifestyle and health-related characteristics. J Epidemiol Community Health. 2016;70:A26–A26.

[28] Mach F, Baigent C, Catapano AL, Koskinas KC, Casula M, Badimon L, et al. 2019 ESC/EAS Guidelines for the management of dyslipidaemias: lipid modification to reduce cardiovascular risk. Eur Heart J. 2020;41:111–88. 31504418 10.1093/eurheartj/ehz455

[29] Duan Y, Gong K, Xu S, Zhang F, Meng X, Han J. Regulation of cholesterol homeostasis in health and diseases: from mechanisms to targeted therapeutics. Signal Transduct Target Ther. 2022;7:265. 35918332 10.1038/s41392-022-01125-5 PMC9344793

[30] Borén J, Taskinen M-R, Björnson E, Packard CJ. Metabolism of triglyceride-rich lipoproteins in health and dyslipidaemia. Nat Rev Cardiol. 2022;19:577–92. 35318466 10.1038/s41569-022-00676-y

[31] Ridker PM. LDL cholesterol: controversies and future therapeutic directions. Lancet. 2014;384:607–17. 25131980 10.1016/S0140-6736(14)61009-6

[32] Li Z, Zhang B, Liu Q, Tao Z, Ding L, Guo B, et al. Genetic association of lipids and lipid-lowering drug target genes with non-alcoholic fatty liver disease. EBioMedicine. 2023;90:104543. 37002989 10.1016/j.ebiom.2023.104543 PMC10070091

[33] GTEx Consortium. The GTEx Consortium atlas of genetic regulatory effects across human tissues. Science. 2020;369:1318–30. 32913098 10.1126/science.aaz1776 PMC7737656

[34] Võsa U, Claringbould A, Westra H-J, Bonder MJ, Deelen P, Zeng B, et al. Large-scale cis- and trans-eQTL analyses identify thousands of genetic loci and polygenic scores that regulate blood gene expression. Nat Genet. 2021;53:1300–10. 34475573 10.1038/s41588-021-00913-z PMC8432599

[35] Emdin CA, Khera AV, Kathiresan S. Mendelian randomization. JAMA. 2017;318:1925. 29164242 10.1001/jama.2017.17219

[36] Burgess S, Butterworth A, Thompson SG. Mendelian randomization analysis with multiple genetic variants using summarized data. Genet Epidemiol. 2013;37:658–65. 24114802 10.1002/gepi.21758 PMC4377079

[37] Bowden J, Davey Smith G, Burgess S. Mendelian randomization with invalid instruments: effect estimation and bias detection through egger regression. Int J Epidemiol. 2015;44:512–25. 26050253 10.1093/ije/dyv080 PMC4469799

[38] Bowden J, Davey Smith G, Haycock PC, Burgess S. Consistent estimation in Mendelian randomization with some invalid instruments using a weighted median estimator. Genet Epidemiol. 2016;40:304–14. 27061298 10.1002/gepi.21965 PMC4849733

[39] Hartwig FP, Davey Smith G, Bowden J. Robust inference in summary data Mendelian randomization via the zero modal pleiotropy assumption. Int J Epidemiol. 2017;46:1985–98. 29040600 10.1093/ije/dyx102 PMC5837715

[40] Burgess S, Foley CN, Allara E, Staley JR, Howson JMM. A robust and efficient method for Mendelian randomization with hundreds of genetic variants. Nat Commun. 2020;11:376 31953392 10.1038/s41467-019-14156-4 PMC6969055

[41] Yu K, Chen X-F, Guo J, Wang S, Huang X-T, Guo Y, et al. Assessment of bidirectional relationships between brain imaging-derived phenotypes and stroke: a Mendelian randomization study. BMC Med. 2023;21:271. 37491271 10.1186/s12916-023-02982-9 PMC10369749

[42] Korthauer K, Kimes PK, Duvallet C, Reyes A, Subramanian A, Teng M, et al. A practical guide to methods controlling false discoveries in computational biology. Genome Biol. 2019;20:118. 31164141 10.1186/s13059-019-1716-1 PMC6547503

[43] Verbanck M, Chen C-Y, Neale B, Do R. Detection of widespread horizontal pleiotropy in causal relationships inferred from Mendelian randomization between complex traits and diseases. Nat Genet. 2018;50:693–8. 29686387 10.1038/s41588-018-0099-7 PMC6083837

[44] Hemani G, Bowden J, Davey SG. Evaluating the potential role of pleiotropy in Mendelian randomization studies. Hum Mol Genet. 2018;27:R195-208. 29771313 10.1093/hmg/ddy163 PMC6061876

[45] Burgess S, Bowden J, Fall T, Ingelsson E, Thompson SG. Sensitivity analyses for robust causal inference from Mendelian randomization analyses with multiple genetic variants. Epidemiology. 2017;28:30–42. 27749700 10.1097/EDE.0000000000000559 PMC5133381

[46] Brion M-JA, Shakhbazov K, Visscher PM. Calculating statistical power in Mendelian randomization studies. Int J Epidemiol. 2013;42:1497–501. 24159078 10.1093/ije/dyt179 PMC3807619

[47] Zhao SS, Alton P, Rogers K, Hughes DM. Statin use, lipids, and 3-hydroxy-3-methyl-glutaryl coenzyme a reductase inhibition on risk of idiopathic pulmonary fibrosis. Clin Ther. 2024;46:79–81. 37978012 10.1016/j.clinthera.2023.10.018

[48] Jiang Y, Chen R, Xu S, Ding Y, Zhang M, Bao M, et al. Endocrine and metabolic factors and the risk of idiopathic pulmonary fibrosis: a Mendelian randomization study. Front Endocrinol (Lausanne). 2023;14:1321576. 38260151 10.3389/fendo.2023.1321576 PMC10801027

[49] Suryadevara V, Ramchandran R, Kamp DW, Natarajan V. Lipid mediators regulate pulmonary fibrosis: potential mechanisms and signaling pathways. Int J Mol Sci. 2020;21:4257. 32549377 10.3390/ijms21124257 PMC7352853

[50] Yan F, Wen Z, Wang R, Luo W, Du Y, Wang W, et al. Identification of the lipid biomarkers from plasma in idiopathic pulmonary fibrosis by Lipidomics. BMC Pulm Med. 2017;17:174. 29212488 10.1186/s12890-017-0513-4 PMC5719761

[51] O’Callaghan M, Duignan J, Tarling EJ, Waters DK, McStay M, O’Carroll O, et al. Analysis of tissue lipidomics and computed tomography pulmonary fat attenuation volume (CTPFAV ) in idiopathic pulmonary fibrosis. Respirology. 2023;28:1043–52. 37642207 10.1111/resp.14582

[52] Grimaudo S, Bartesaghi S, Rametta R, Marra F, Margherita Mancina R, Pihlajamäki J, et al. PCSK9 rs11591147 R46L loss-of-function variant protects against liver damage in individuals with NAFLD. Liver Int. 2021;41:321–32. 33091218 10.1111/liv.14711

[53] Ning L, Zou Y, Li S, Cao Y, Xu B, Zhang S, et al. Anti-PCSK9 treatment attenuates liver fibrosis via inhibiting hypoxia-induced autophagy in hepatocytes. Inflammation. 2023;46:2102–19. 37466835 10.1007/s10753-023-01865-8 PMC10673768

[54] Bao H, Wang X, Zhou H, Zhou W, Liao F, Wei F, et al. PCSK9 regulates myofibroblast transformation through the JAK2/STAT3 pathway to regulate fibrosis after myocardial infarction. Biochem Pharmacol. 2024;220:115996. 38154546 10.1016/j.bcp.2023.115996

[55] Wu D, Zhou Y, Pan Y, Li C, Wang Y, Chen F, et al. Vaccine against PCSK9 improved renal fibrosis by regulating fatty acid β-oxidation. J Am Heart Assoc. 2020;9:e014358. 31870234 10.1161/JAHA.119.014358 PMC6988173

[56] Lee C, Kwak SH, Han J, Shin JH, Yoo B, Lee YS, et al. Repositioning of ezetimibe for the treatment of idiopathic pulmonary fibrosis. Eur Respir J. 2024;63:2300580. 38359963 10.1183/13993003.00580-2023 PMC11096666

