# Supplementary figures and images for: Exploring the causal effect between lipid-modifying drugs and idiopathic pulmonary fibrosis: a drug-target Mendelian randomization study
Source: Lipids Health Dis. 2024 Aug 1;23:237. doi: 10.1186/s12944-024-02218-6 (PMC11293199; doi:10.1186/s12944-024-02218-6)

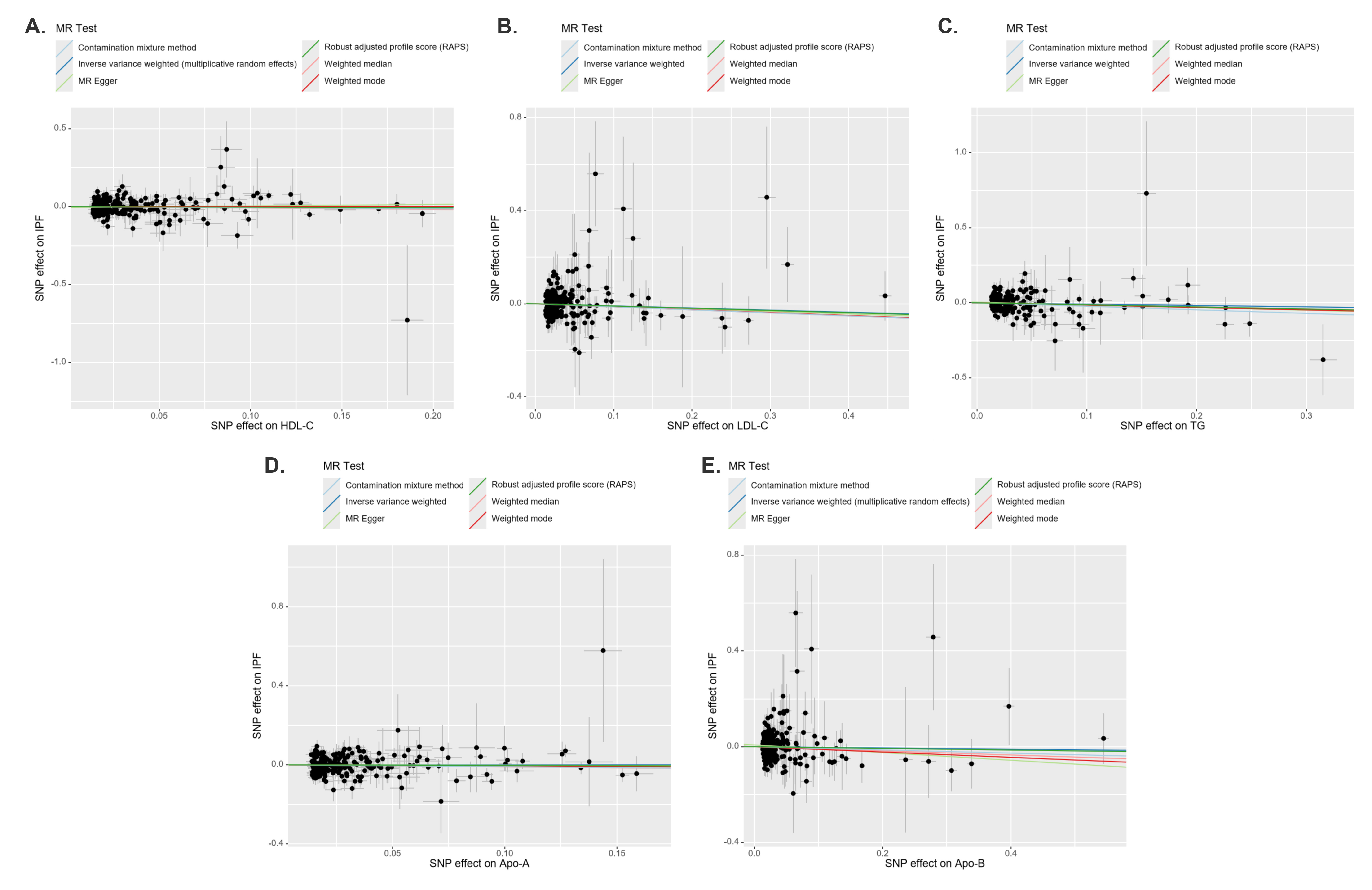

Supplement: Supplementary file 2 — Additional file 2: Fig. S1. Scatter plots of the association between lipid traits and IPF; A. High-density lipoprotein cholesterol on idiopathic pulmonary fibrosis; B. Low-density lipoprotein cholesterol on idiopathic pulmonary fibrosis; C. Triglyceride on idiopathic pulmonary fibrosis; D. Apolipoprotein A on idiopathic pulmonary fibrosis; E. Apolipoprotein B on idiopathic pulmonary fibrosis. [file 12944_2024_2218_MOESM2_ESM.jpeg]

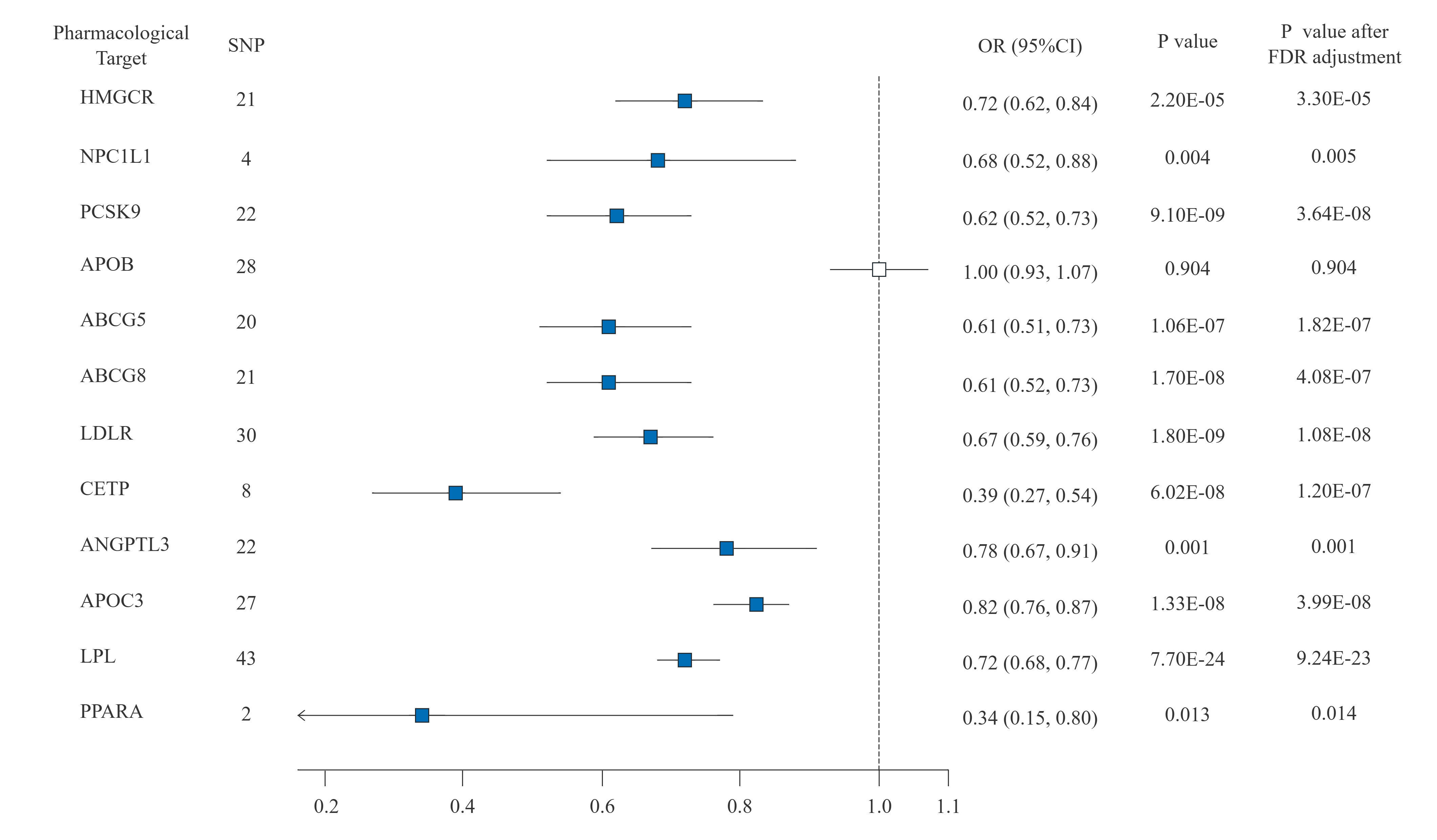

Supplement: Supplementary file 3 — Additional file 3: Fig. S2. Forest plots of the association between genetically proxied lipid-modifying drug and CHD risk. [file 12944_2024_2218_MOESM3_ESM.jpeg]

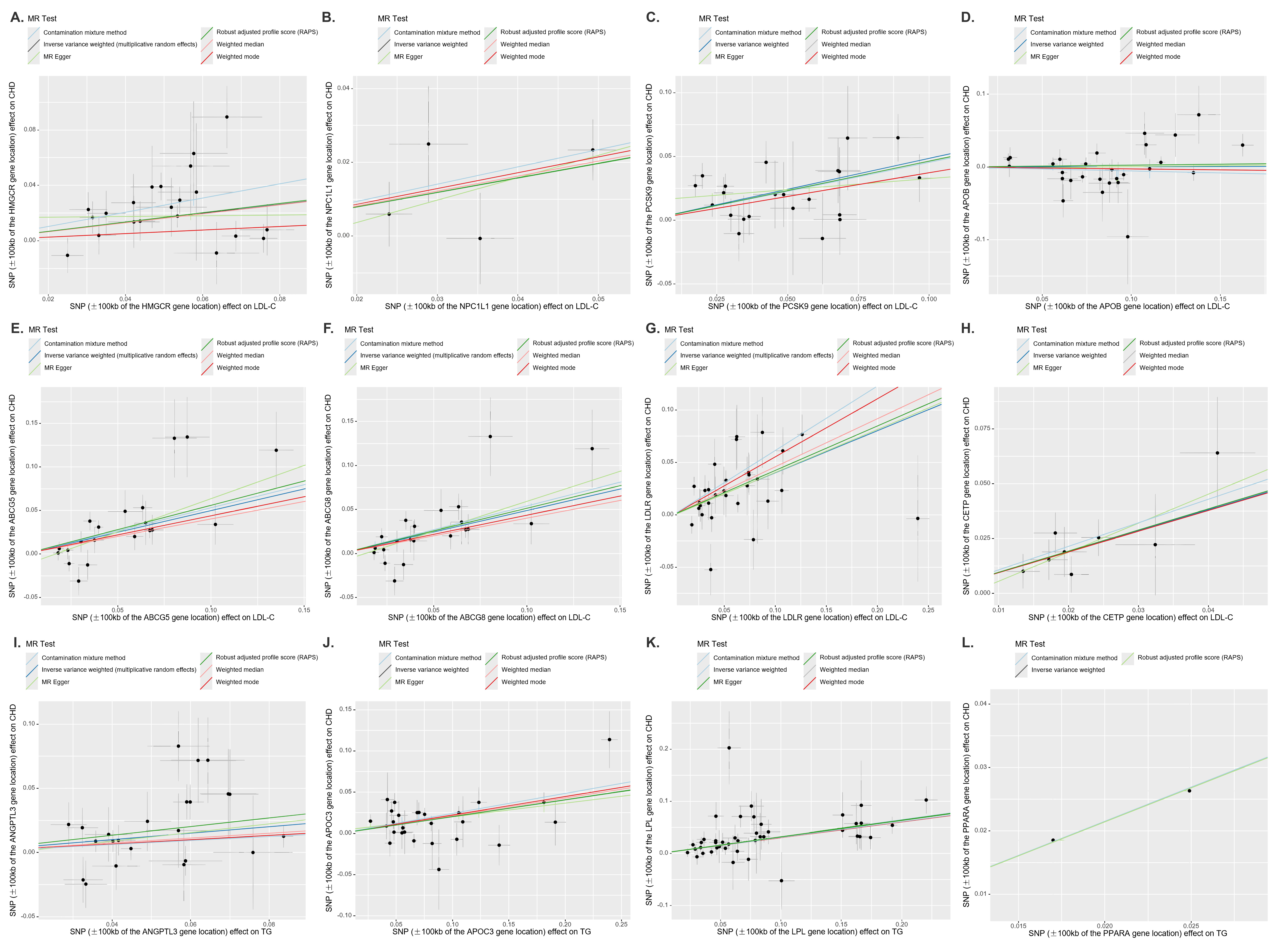

Supplement: Supplementary file 4 — Additional file 4: Fig. S3. Scatter plots of the association between genetically proxied lipid-modifying gene targets and CHD. A. HMGCR on coronary heart disease; B. NPC1L1 on coronary heart disease; C. PCSK9 on coronary heart disease; D. APOC on coronary heart disease; E. ABCG5 on coronary heart disease; F. ABCG8 on coronary heart disease; G. LDLR on coronary heart disease; H. CETP on coronary heart disease; I. ANGPTL3 on coronary heart disease; J. APOC3 on coronary heart disease; K. LPL on coronary heart disease; L. PPARA on coronary heart disease. [file 12944_2024_2218_MOESM4_ESM.jpg]

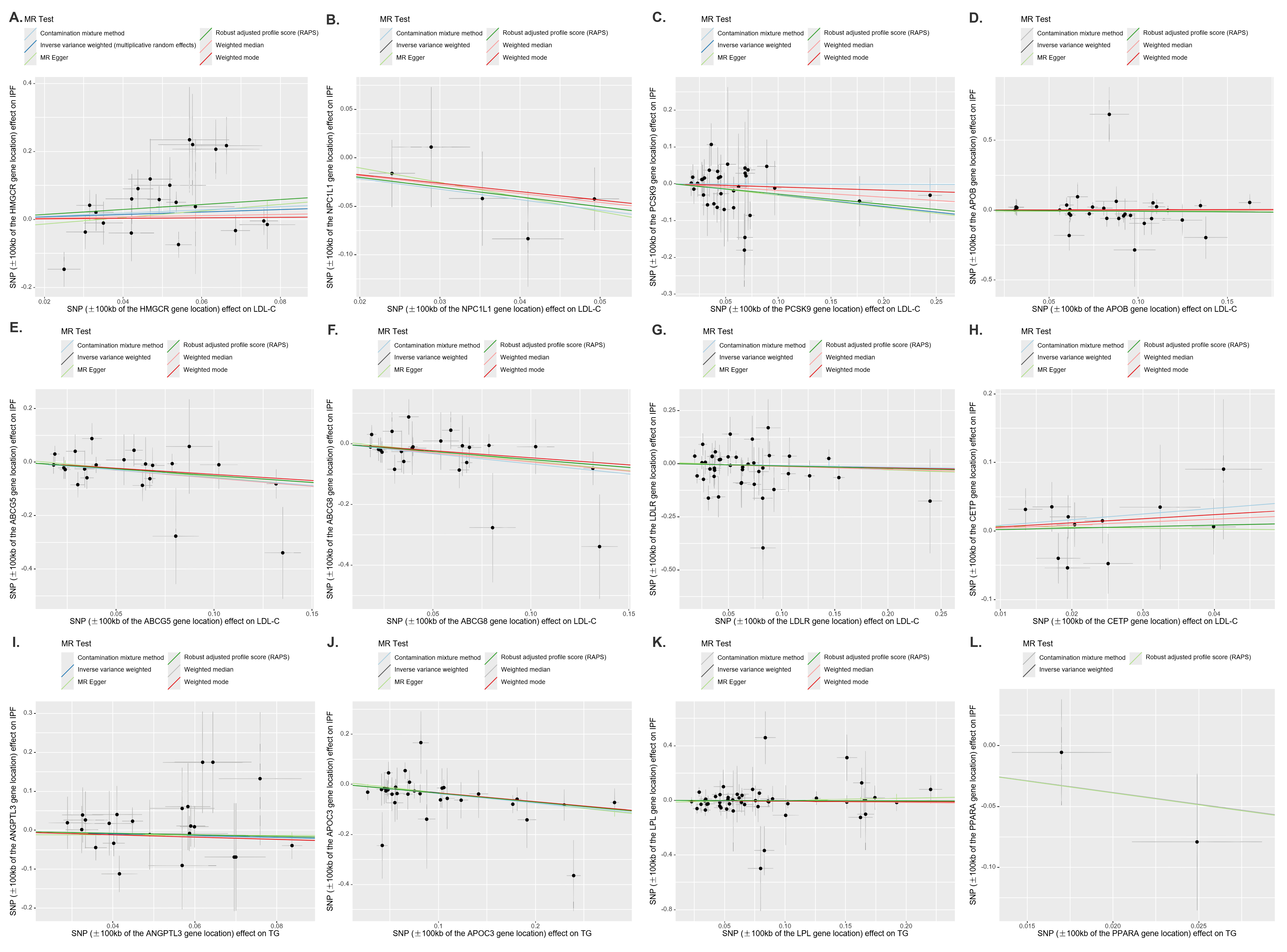

Supplement: Supplementary file 5 — Additional file 5: Fig. S4. Scatter plots of the association between genetically proxied lipid-modifying gene targets and IPF. A. HMGCR on idiopathic pulmonary fibrosis; B. NPC1L1 on idiopathic pulmonary fibrosis; C. PCSK9 on idiopathic pulmonary fibrosis; D. APOC on idiopathic pulmonary fibrosis; E. ABCG5 on idiopathic pulmonary fibrosis; F. ABCG8 on idiopathic pulmonary fibrosis; G. LDLR on idiopathic pulmonary fibrosis; H. CETP on idiopathic pulmonary fibrosis; I. ANGPTL3 on idiopathic pulmonary fibrosis; J. APOC3 on idiopathic pulmonary fibrosis; K. LPL on idiopathic pulmonary fibrosis; L. PPARA on idiopathic pulmonary fibrosis. [file 12944_2024_2218_MOESM5_ESM.jpg]

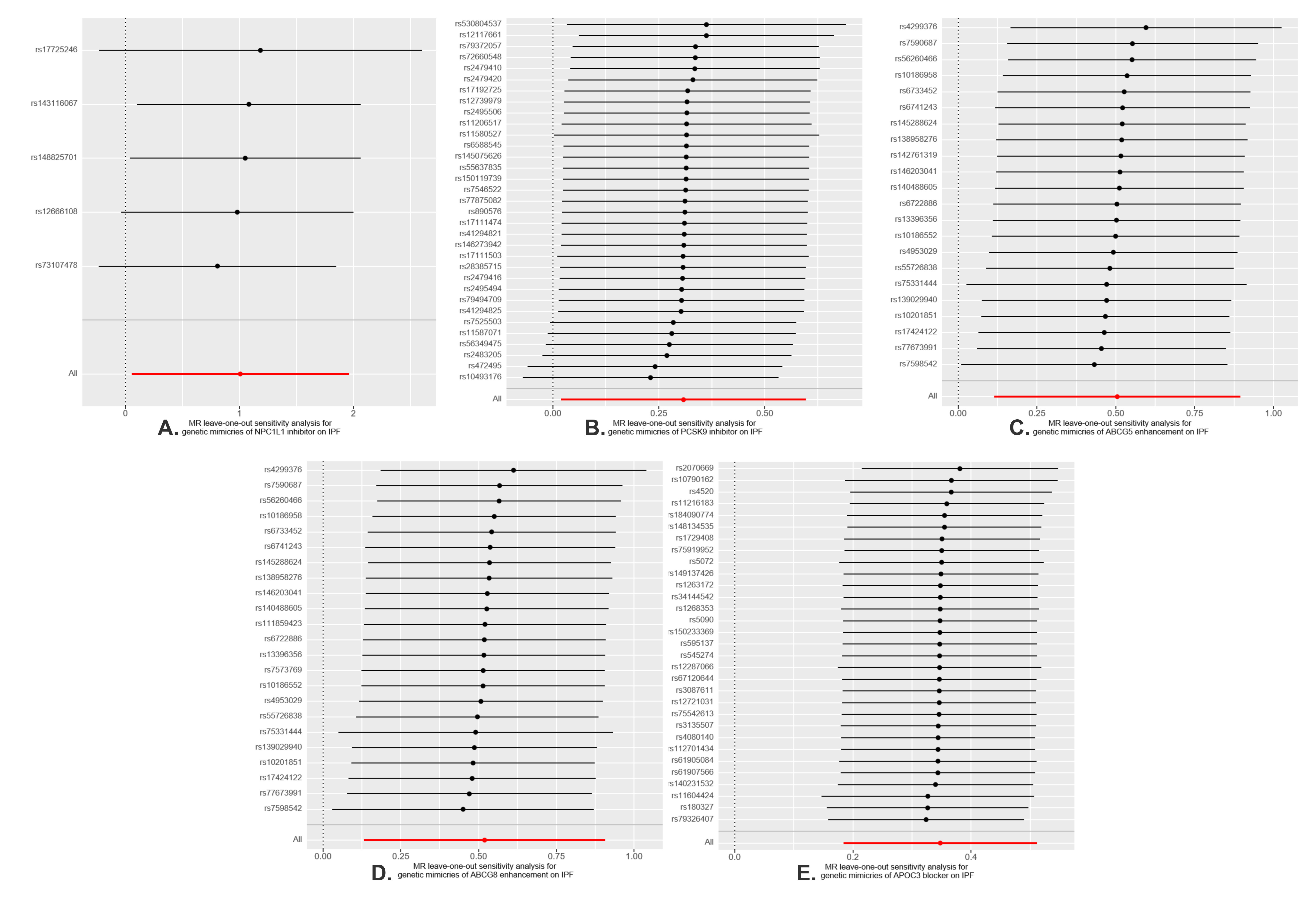

Supplement: Supplementary file 6 — Additional file 6: Fig. S5. Plots of “leave-one-out” analyses for MR analyses of the causal effect of lipid-modifying drugs on IPF using primary effect. A. Genetic mimicries of NPC1L1 inhibitor on idiopathic pulmonary fibrosis; B. Genetic mimicries of PCSK9 inhibitor on idiopathic pulmonary fibrosis; C. Genetic mimicries of ABCG5 enhancement on idiopathic pulmonary fibrosis; D. Genetic mimicries of ABCG8 enhancement on idiopathic pulmonary fibrosis; E. Genetic mimicries of APOC3 blocker on idiopathic pulmonary fibrosis. [file 12944_2024_2218_MOESM6_ESM.jpeg]

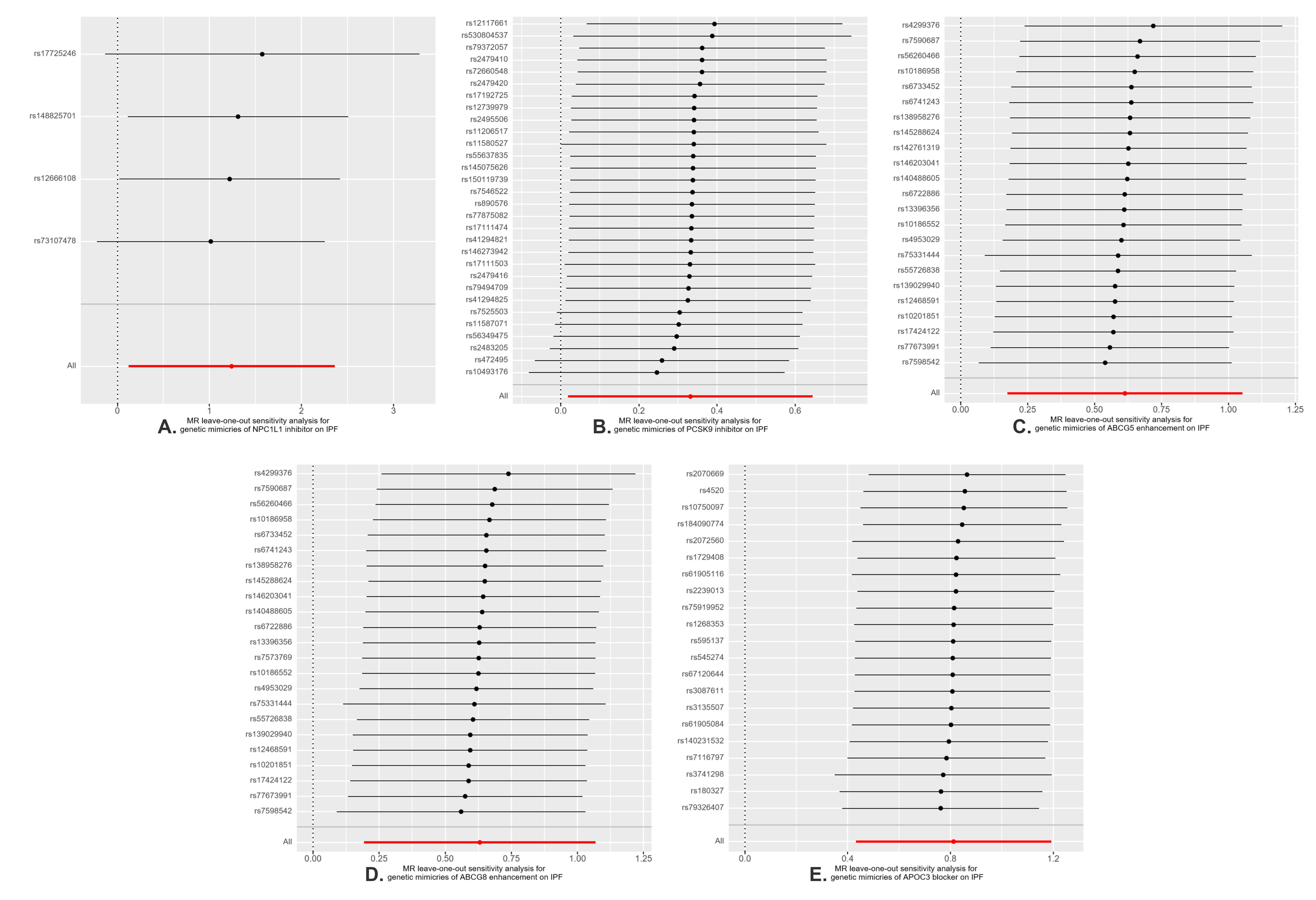

Supplement: Supplementary file 7 — Additional file 7: Fig. S6. Plots of “leave-one-out” analyses for MR analyses of the causal effect of lipid-modifying drugs on IPF using alternative effect. A. Genetic mimicries of NPC1L1 inhibitor on idiopathic pulmonary fibrosis; B. Genetic mimicries of PCSK9 inhibitor on idiopathic pulmonary fibrosis; C. Genetic mimicries of ABCG5 enhancement on idiopathic pulmonary fibrosis; D. Genetic mimicries of ABCG8 enhancement on idiopathic pulmonary fibrosis; E. Genetic mimicries of APOC3 blocker on idiopathic pulmonary fibrosis. [file 12944_2024_2218_MOESM7_ESM.jpeg]
